# Intake of myo-inositol hexaphosphate and urinary excretion of inositol phosphates in Wistar rats: Gavage *vs*. oral administration with sugar

**DOI:** 10.1371/journal.pone.0223959

**Published:** 2019-10-18

**Authors:** F. Grases, A. Costa-Bauzá, F. Berga, R. M. Gomila, G. Martorell, M. R. Martínez-Cignoni

**Affiliations:** 1 Laboratory of Renal Lithiasis Research, University Institute of Health Sciences Research (IUNICS-IdISBa), University of Balearic Islands, Ctra Valldemossa, Palma de Mallorca, Spain; 2 Serveis Cientificotècnics, University of Balearic Islands, Ctra Valldemossa, Palma de Mallorca, Spain; 3 Grup de Metabolisme Energètic i Nutrició, Dept. Biologia Fonamental i Ciències de la Salut, University Institute of Health Sciences Research (IUNICS-IdISBa), University of Balearic Islands, Ctra Valldemossa, Palma de Mallorca, Spain; University of Illinois, UNITED STATES

## Abstract

**Objective:**

To evaluate the urinary levels of inositol phosphates (InsPs) in rats that received different salts of myo-inositol hexaphosphate (InsP6) by gavage or by oral administration.

**Methods:**

Thirty rats received AIN-76A diet (in which InsPs are undetectable) for 15 days. Then, 12 rats received InsP6 by gavage as a Na salt or a Ca/Mg salt; after 4 days, the Na or Ca/Mg InsP6 was administered with water containing 15 g/L sucrose and urine samples were collected. The other 18 rats received oral InsP6, in which 0.5 g of sugar was combined with InsP6 as a Na salt, a Ca/Mg salt, or a Na salt with CaCO_3_; daily urine samples were collected. Urine levels of InsPs were determined using a nonspecific method and a specific method (polyacrylamide gel electrophoresis, PAGE), and different InsPs were identified by mass spectroscopy (MS).

**Results:**

After 15 days of the InsP6-free diet, the non-specific method detected no urinary InsPs, and MS detected only InsP2. After administration of Na-InsP6 by gavage, the non-specific method indicated more urinary InsPs than the amount of InsP6 determined by PAGE. MS indicated the presence of urinary InsP2, InsP3, InsP4, InsP5, and InsP6 in these rats, with notable variations among animals. Use of the same treatment to administer Ca/Mg-InsP6 led to a lower overall content of urinary InsPs and a lower level of InsP6. Oral administration of InsP6 as a sugar pill led to lower urinary levels of InsPs than administration of InsP6 by gavage, and administration as a Ca/Mg pill or a Ca/Mg pill with CaCO_3_ led to lower levels than administration as a Na pill.

**Conclusion:**

Administration of InsP6 to rats leads to the excretion of a mixture of different InsPs. Rats more effectively absorb InsP6 when supplied without dietary components that interfere with its uptake, such as the Ca ion and sugar.

## Introduction

Some components of the Mediterranean diet, such as nuts, legumes, and whole grains, contain abundant myo-inositol hexaphosphate (InsP6), and some of the positive effects of this diet are thus attributed to this compound [1]. The metabolic transformation of InsP6 must be characterized to better understand its beneficial effects, but few studies have examined this topic. Recent studies showed that after InsP6 appears in the body's fluids, several phosphatases metabolize it into multiple phosphorylated forms, from InsP5 to InsP2 [2]. In addition, InsP6 also forms within cells by *de novo* synthesis [3].

Studies of InsP6 and its metabolism are also complicated because of the analytical complexity of identification and quantification of the different species and isomers. One reason for this is that the poor spectral characteristics of these molecules make it difficult to identify by traditional spectrophotometric or fluorometric methods [1]. A second reason is that they are present at very low concentrations in media that contains abundant phosphate ion, and they are readily adsorbed onto different materials (such as steel [4], [5]) so during isolation, recovery can be poor or they can be transformed to different forms [6]. However, the coupling of high performance liquid chromatography (HPLC) with mass spectrometry (MS) has improved the detection of these compounds [7]. HPLC coupled to tandem mass spectrometry (MS/MS) has led to further improvements in the measurement of inositol phosphates (InsPs) in biological samples [8], [9].

Because of the analytical difficulties in measuring InsPs, there is limited knowledge of the influence of the dietary InsP6, the absorption and metabolism of InsP6, and the urinary excretion of InsPs. In this paper, we examined the absorption, metabolism, and excretion of InsP6 in female Wistar rats.

## Materials and methods

### Animals, diets and experimental design

Female Wistar rats, which were purchased from Envigo (Barcelona, Spain), were used to evaluate urinary excretion of InsPs after oral administration of different formulations of InsP6. The animals were housed in individual cages in a temperature-controlled room (23 +/- 1ºC) with a 12h light-dark cycle, and received an AIN-76A diet (Harlan, Indianapolis, IN, USA), a synthetic purified food that has undetectable levels of InsP6 and other InsPs. The experimental interventions began 15 days after rats began consuming this diet.

### Administration of InsP6 by gavage

Twelve female rats were weighed and separated into two groups (6 animals per group) so that the average rat weight in each group was similar (260 g). The Na-InsP6 gavage group received gavage of 0.03 mmol of InsP6Na_12_ (27.7 mg/1.3mL) each morning (Group A) and the Ca/Mg-InsP6 gavage group received gavage of 0.03 mmol InsP6Mg_2_Ca_4_ (25.71 mg/1.3mL) each morning (Group B). After day 4 (4 administrations), the gavage halted and InsP6 was administered by a bottle with 15 mL of water containing 0.03 mmol of Na-InsP6 or Ca/Mg-InsP6 and 15 g/L of sucrose for 3 and 10 days. On day 7 (4 days gavage + 3 days liquid) and day 14 (4 days gavage + 10 days liquid), 24 h urine was collected using individual metabolic cages. The rats consumed tap water with 5 g/L sucrose during these 24 h periods.

### Oral administration of InsP6 as a sugary pellet

Eighteen animals were divided into three groups (6 animals per group) so that the average rat weight in each group was 240 g. Each morning, the Na-InsP6 pill group received 0.03 mmol InsP6Na_12_ mixed with 0.5 g of fondant (semi-solid sugary mixture, as vehicle) (Group C) [10], [11], the Ca/Mg-InsP6 pill group received 0.03 mmol InsP6Mg_2_Ca_4_ mixed with 0.5 g of fondant (Group D), and the Na-InsP6-Ca pill group received 0.03 mmol InsP6Na_12_ + 0.36 mmol CaCO_3_ mixed with 0.5 g fondant (Group E). Then 24 h urine samples were collected on days 0, 7, 14, 21, 28, and 35 using individual metabolic cages.

All procedures were carried out according to directive 86/609/EEC, regarding the protection of animals used for experimental and other scientific purposes. Official permission to perform the animal experiments was provided by the Comité de Ética de Experimentación Animal (CEEA) of the University of Balearic Islands (Exp. 2016/19/AEXP).

### Non specific spectrometric quantification of InsPs

Global InsPs quantification was performed by an indirect colorimetric method, using Aluminum-xylenol orange [12].

### Extraction of InsPs with TiO2

The extraction of InsPs was performed as previously described [2, 13], using TiO_2_ (Titansphere TiO_2_ 5 μm; GL Sciences).

### Identification of InsPs by MS

**Fig 1:**
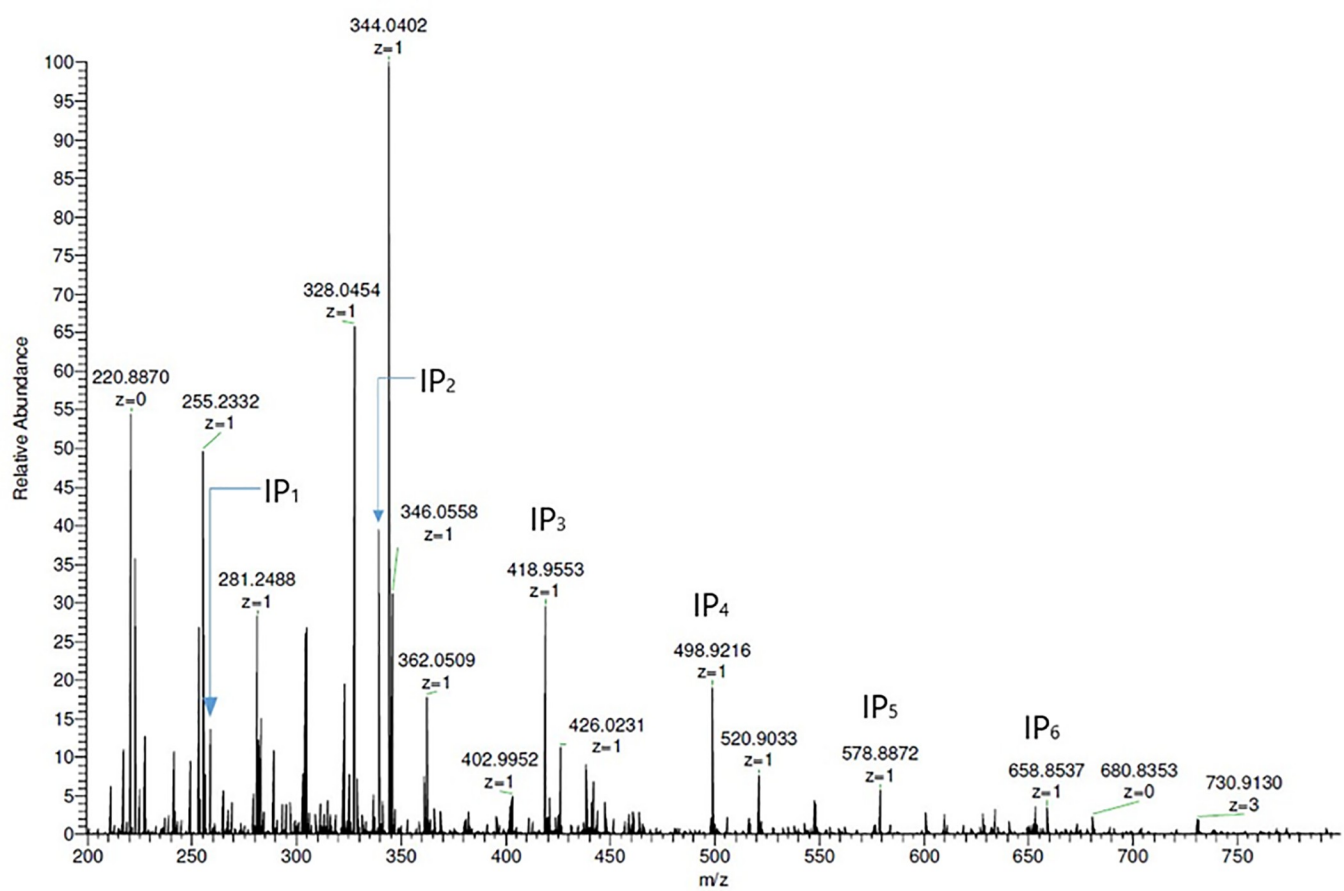
Mass spectrum of a eluate prepared using TiO_2_ purification. Rat # 6 (Group A) after 14 days of IP6Na_12_ administration.

After InsPs extraction with TiO_2_, a total of 150 μL of the reconstituted eluate was separated, and 10 μL was injected into a Q Exactive Orbitrap high-resolution mass spectrometer that had a heated electrospray ionization (HESI) probe (Thermo Fisher Scientific, Waltham, USA), which was operated in negative ionization mode. An example of Mass spectrum is provided in Fig 1.

### Identification and semi-quantification of InsP6 by PAGE

After InsPs extraction with TiO_2_, a volume of 850 μL of the reconstituted eluate was evaporated until the volume was 30 μL, for use in polyacrylamide gel electrophoresis (PAGE), following a previously described procedure [14].

### Statistics

Data are presented as means ± standard error (SE). Intragroup comparison of urinary concentration and excretion of InsPs (at different days) were performed using one–way repeated measures ANOVA and Bonferroni as post hoc test for each group. Intergroup comparisons of urinary concentration and excretion of InsPs at days 0, 7, 14, 21, 28 and 35 were assessed using two-way repeated measures ANOVA in order to evaluate effect of treatment, periods and interaction treatment x period. A two-tailed *p*-value less than 0.05 was considered statistically significant. Statistical analyses were performed using SPSS version 23.0 (SPSS Inc., Chicago, IL, USA).

## Results

### Administration of InsP6 by gavage

**Fig 2:**
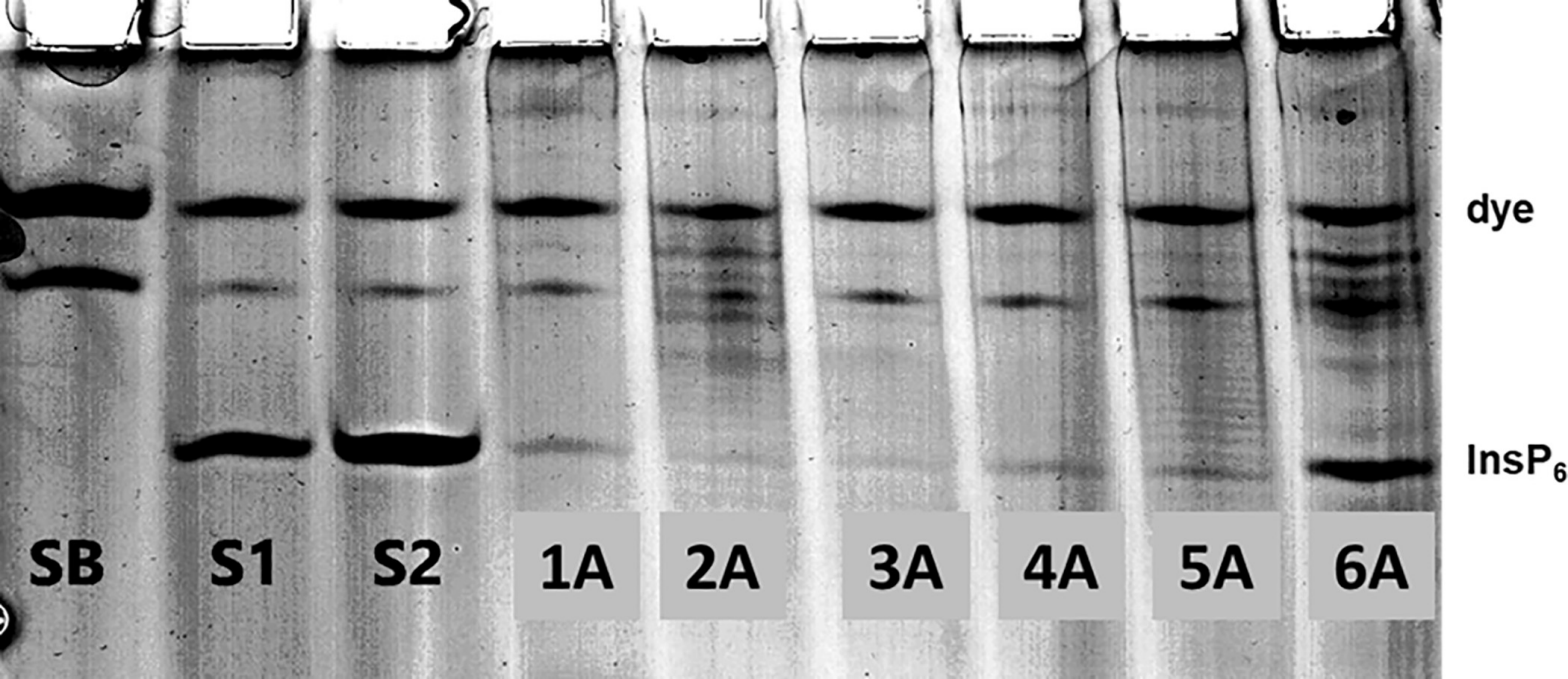
Detection of InsP6 in urine of rat by PAGE, after 14 days of InsP6Na_*12*_ administration (Group A, day 14). SB (sample Buffer), S1 (Standard 1: 1.25nmols InsP6), S2 (Standard 2: 5 nmols InsP6), urine samples (rats 1A, 2A, 3A, 4A, 5A, 6A).

**Fig 3:**
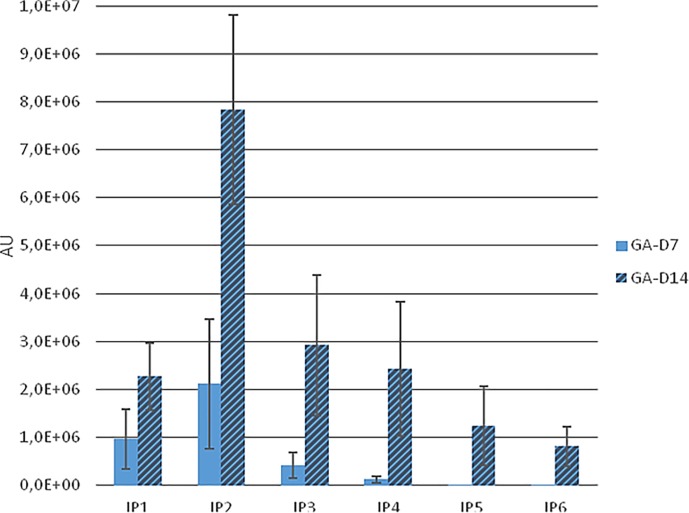
Identification of InsPs by Mass Spectrometry (MS) after 7 and 14 days of administration of InsP6Na_*12*_ (Group A). Mean ± SE of the values obtained by each rat.

In agreement with previous experiments [2], when rats received an InsP6-free diet for several weeks, a non-specific method detected no InsPs, but MS detected InsP2. Administering Na-InsP6 by gavage plus 3/10 days with water containing Na-InsP6 and sucrose, we observed the presence of urinary InsP6 at variable amounts (Fig 2). As expected, the total InsPs determined by the non-specific method was greater than the amount of InsP6 determined by PAGE (Tables 1–3). The MS data indicated a large increase of urinary InsP2, and the presence of InsP3, InsP4, InsP5, and InsP6 (Fig 3). There were also notable variations among the animals.

**Table 1:** Concentration and excretion values of InsP6 obtained by semiquantification with polyacrylamide gel electrophoresis (PAGE). During the collection day rats drank Tap Water with 5g/L of sucrose to increase the diuresis. Group A–administration of IP6Na_12_, Group B–administration of InsP6Mg_2_ Ca_4._ Values are expressed as mean ± SE. Comparison between groups were performed by independent samples t-test.

	*GROUP A* *(day 14)*	*GROUP B* *(day 14)*	*p-value*
**[InsP**_**6**_**] μM**			
***1***	0.043	0.016	
***2***	0.030	0.018	
***3***	0.025	0.013	
***4***	0.119	0.024	
***5***	0.069	0.013	
***6***	0.266	0.027	
***Mean* ± *SE***	0.092 ± 0.038	0.018 ± 0.002	0.108
**Exc InsP**_**6**_ **nm/20h**			
***1***	0.492	0.445	
***2***	0.435	0.275	
***3***	0.331	0.632	
***4***	0.831	0.327	
***5***	0.963	0.370	
***6***	4.929	0.322	
***Mean* ± *SE***	1.330 ± 0.727	0.395 ± 0.053	0.255

**Table 2:** Results of concentration obtained by non-specific spectrometric quantification of InsPs. Group A–administration of IP6Na_12_ by gavage Group B–administration of InsP6Mg_2_ Ca_4._by gavage Group C–oral administration of IP6Na_12_, Group D–oral administration of IP6Mg_2_Ca_4_, Group E–oral administration of IP6Na_12_ + Ca. Values are expressed as mean ± SE.

	*Group A*	*Group B*	*Group C*	*Group D*	*Group E*	*Intergroup p-value*
[InsPs] μM						
Day 0	0.047 ± 0.031	0.138 ± 0.100	0.014 ± 0.006	0.082 ± 0.010	0.039 ± 0.018	0.404
Day 7	0.530 ± 0.047 ^1^	0.162 ± 0.055 ^a^	0.063 ± 0.043 ^a^	0.051 ± 0.046 ^a^	0.076 ± 0.016 ^a^	<0.001
Day 14	0.335 ± 0.066 ^1^	0.238 ± 0.040	0.216 ± 0.195	0.036 ± 0.036 ^a^	0.188 ± 0.099	0.032
Day 21			0.519 ± 0.134 ^1^^,^^3^	0.190 ± 0.061 ^3^	0.017 ± 0.017 ^b^	0.012
Day 28			0.173 ± 0.050 ^1^^,^^4^	0.072 ± 0.031 ^4^	0.279 ± 0.098 ^1^^,^^3^^,^^c^	0.007
Day 35			0.663 ± 0.317	0.167 ± 0.049 ^2^^,^^3^	0.555 ± 0.175 ^1^^,^^3^	0.060

Intragroup comparisons were analyzed using one-way repeated measures ANOVA for each group. Intergroup comparisons were performed by two-way repeated measures ANOVA (at 0, 7, 14, 21, 28 and 35 days).

^1^: p<0.05 vs. corresponding value at day 0 (intragroup)

^2^: p<0.05 vs. corresponding value at day 7 (intragroup)

^3^: p<0.05 vs. corresponding value at day 14 (intragroup)

^4^: p<0.05 vs. corresponding value at day 21 (intragroup)

^a^: p<0.05 vs. corresponding value of group A (intergroup)

^b^: p<0.05 vs. corresponding value of group C (intergroup)

^c^: p<0.05 vs. corresponding value of group D (intergroup)

**Table 3:** Excretion values obtained by non-specific spectrometric quantification of InsPs. Group A–administration of IP6Na_12_ by gavage Group B–administration of InsP6Mg_2_ Ca_4._by gavage Group C–oral administration of IP6Na_12_, Group D–oral administration of IP6Mg_2_Ca_4_, Group E–oral administration of IP6Na_12_ + Ca. Values are expressed as mean ± SE.

	*Group A*	*Group B*	*Group C*	*Group D*	*Group E*	*Intergroup p-value*
[InsPs] Exc (nmol/20h)						
Day 0	0.320 ± 0.131	0.910 ± 0.311	0.565 ± 0.236	2.553 ± 0.423 ^a^^,^^b^^,^^c^	0.920 ± 0.379 ^d^	<0.001
Day 7	6.925 ± 1.279 ^1^	2.398 ± 0.537 ^1^^,^^a^	0.963 ± 0.313 ^a^	0.893 ± 0.704 ^a^	1.611 ± 0.401 ^a^	<0.001
Day 14	4.698 ± 1.507 ^1^	4.840 ± 0.417 ^1^^,^^2^	0.465 ± 0.329 ^a^^,^^b^	0.199 ± 0.199 ^1^^,^^a^^,^^b^	1.551 ± 0.441 ^a^	<0.001
Day 21			4.114 ± 0.335 ^1^^-^^3^	3.751 ± 0.391 ^1^^-^^3^	0.447 ± 0.447 ^c^^,^^d^	0.001
Day 28			2.311 ± 0.365 ^1^^-^^3^	1.373 ± 0.081 ^3^^,^^4^	2.818 ± 0.391 ^1^^,^^3^	0.125
Day 35			6.369 ± 0.416 ^1^^-^^5^	1.226 ± 0.213 ^1^^,^^3^^,^^4^^,^^c^	4.827 ± 0.574 ^1^^-^^5^^,^^d^	<0.001

Intragroup comparisons were analyzed using one-way repeated measures ANOVA for each group. Intergroup comparisons were performed by two-way repeated measures ANOVA (at 0, 7, 14, 21, 28 and 35 days).

^1^: p<0.05 vs. corresponding value at day 0 (intragroup)

^2^: p<0.05 vs. corresponding value at day 7 (intragroup)

^3^: p<0.05 vs. corresponding value at day 14 (intragroup)

^4^: p<0.05 vs. corresponding value at day 21 (intragroup)

^5^: p<0.05 vs. corresponding value at day 28 (intragroup)

^a^: p<0.05 vs. corresponding value of group A (intergroup)

^b^: p<0.05 vs. corresponding value of group B (intergroup)

^c^: p<0.05 vs. corresponding value of group C (intergroup)

^d^: p<0.05 vs. corresponding value of group D (intergroup)

**Fig 4:**
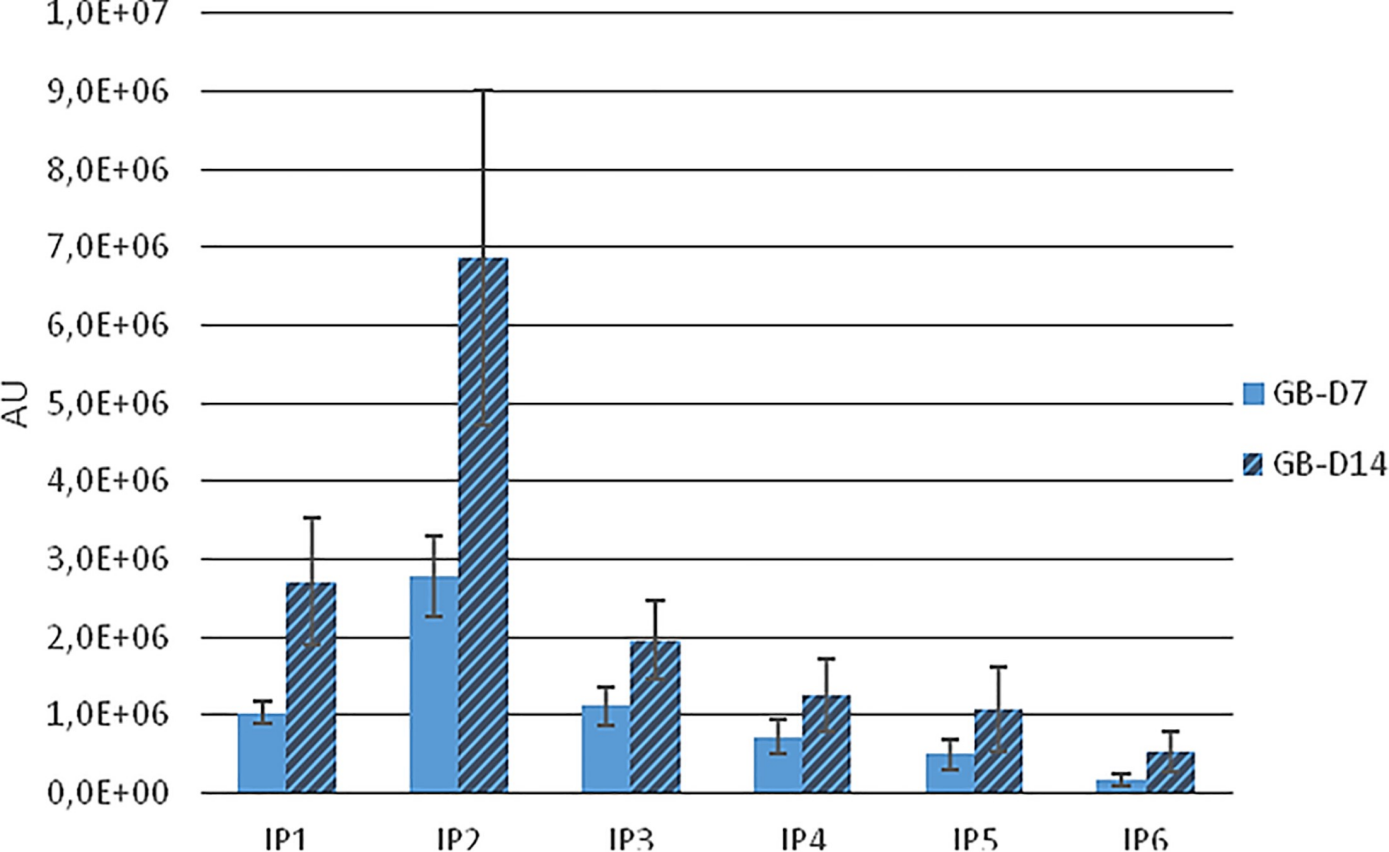
Identification of InsPs by Mass Spectrometry (MS) after 7 and 14 days of administration of InsP6Mg_*2*_Ca_*4*_ (Group B). Mean±SE of the values obtained by each rat.

When the same procedure was performed using Ca/Mg-InsP6, the urinary levels of overall InsPs and specific InsP6 (determined by PAGE) were lower (Tables 1–3). The MS data indicated the presence of InsP2, InsP3, InsP4, InsP5 and InsP6, but in smaller amounts than in rats given Na-InsP6 (Fig 4).

### Administration of InsP6 as a sugar pill

**Fig 5:**
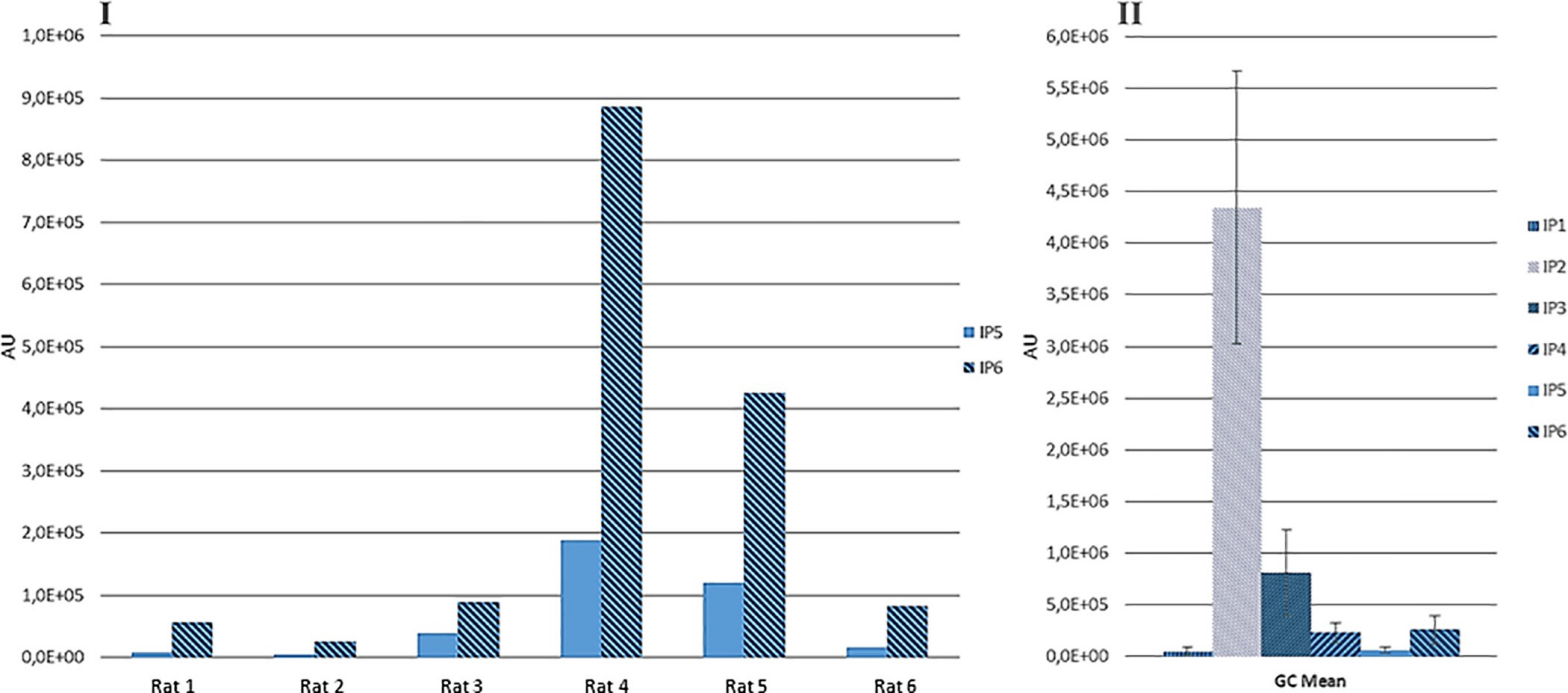
Identification of InsPs by Mass Spectrometry (MS) after 21 days of administration of InsP6Na_*12*_ without calcium (Group C). InsP5 and InsP6 values obtained by each rat of the group (I) and the Mean±SE of InsP1-InsP6 (II).

When Na-InsP6 was administered as a sugary pill (rather than gavage) for the same period of time, there were lower amounts of urinary InsPs (Tables 2 and 3). In addition, the amounts were even lower when InsP6 was administered as Ca/Mg-InsP6 or as Na-InsP6-Ca rather than as a Na salt (Tables 2 and 3). Interestingly, the amounts of InsPs gradually increased during the 35 days of follow-up (Tables 2 and 3). The MS results indicated the presence of InsP6 in very variable amounts (Fig 5).

## Discussion

A previous study reported that when rats consumed an InsP6-free diet for a long period, only InsP2 was detected in their urine [2], as has been observed in the present study. The results presented here indicated that the excretion of InsPs was much greater when InsP6 was administered alone (by gavage) as Na-InsP6 than as Ca/Mg-InsP6, as can be seen in Tables 2 and 3. The post-gavage administration of InsP6 in drinking water with sucrose did not increase the amount of urinary InsPs. The specific determination of InsP6 by PAGE and MS detected InsP6 in urinary samples at 14 days of rats that received Na-InsP6 by gavage. Administration of Ca/Mg-InsP6 by gavage led to lower urinary levels of InsP6, InsP5, InsP4, and InsP3 than administration of Na-InsP6 by gavage.

Administration of InsP6 as a pill with 0.5 g fondant, rather than by gavage, led to much lower amounts of urinary InsPs. Despite this, the InsPs increased very gradually over 35 days in rats given InsP6 as a sugary pill. The amounts of urinary InsP6 were lower in rats given InsP6 as a sugary pill than by gavage.

As in previous studies [12], during InsP6 administration we initially observed a notable increase in the urinary level of InsPs, and this was followed by a decrease. This is probably due to the induction of metabolic enzymes over time. Normally, urinary excretion of large amounts of total InsPs accompanies an increased excretion of InsP6, although this correlation does not always occur. We also observed that when the urinary levels of total InsPs are low, urinary InsP6 was only detected in some of the animals, although the other InsPs were present in all animals. This may be due actual biological variations among animals (probably as a consequence of different phytase and alkaline phosphatase levels), or the result of an analytical problem caused by the complexities of measuring InsP6 in biological samples. Thus, it is necessary to consider that some of the benefits from InsP6 consumption are not exclusively due to InsP6, and that other InsPs can also provide important effects. For example, InsP6 and/or mixtures of its hydrolysis products can both act as potent crystallization inhibitors and prevent the development of pathological calcifications [15, 16].

The present study also shows that the presence of sugar or the calcium ion seems to hinder the absorption of InsP6, thus resulting in reduced excretion. In fact the calcium ion forms an insoluble InsP6 salt and the sugar forms insoluble adducts that prevent absorption. In this way, InsP6 is similar to bisphosphonates, in that maximum absorption occurs with sodium or potassium salt and fasting, and other nutrients hinder its absorption.

A limitation of the present study is that we administered gavage of InsP6 for a short period. This was unavoidable because repeated gavage over many days stresses the animals. Another limitation is that our identification of the different InsPs by MS should be considered tentative. Although our identification of InsP6 is certain, we cannot be certain of its quantitation, nor of the quantitation of other InsPs, because breakage processes of these compounds may have occurred during the detection process.

## Conclusion

Dietary consumption of InsP6 leads to the excretion of a mixture of InsPs, from InsP2 to InsP6. The urinary level of InsPs excreted correlates with the amount of InsP6 present. Absorption of InsP6 is greater when it is supplied in the absence of other dietary components, such as calcium ion or sugar, which interfere with uptake.

## Supporting information

S1 Table Results of concentration and excretion determined by non-specific spectrometric quantification of InsPs. During the collection day rats drank Tap Water with 5g/L of sucrose to increase the diuresis. Group A–administration of IP6Na_12_, Group B–administration of phytin.
(PDF)

S2 Table Concentration and excretion values of InsP6 obtained by semiquantification with polyacrylamide gel electrophoresis (PAGE). During the collection day rats drank Tap Water with 5g/L of sucrose to increase the diuresis. Group A–administration of IP6Na_12_, Group B–administration of phytin.
(PDF)

S3 Table Results of concentration obtained by non-specific spectrometric quantification of InsPs. During the collection day rats drank Tap Water with 10 g/L of sucrose to increase the diuresis. GC–administration of IP6Na_12_, GD–administration of IP6Mg_2_Ca_4_, GE–IP6Na_12_ + Ca.
(PDF)

S4 Table Excretion values obtained by non-specific spectrometric quantification of InsPs. During the collection day rats drank Tap Water with 10g/L of sucrose to increase the diuresis. GC–administration of IP6Na_12_, GD–administration of IP6Mg_2_Ca_4_, GE–IP6Na_12_ + Ca.
(PDF)
