# Supplementary material for: Intake of myo-inositol hexaphosphate and urinary excretion of inositol phosphates in Wistar rats: Gavage vs. oral administration with sugar
Source: PLoS One. 2019 Oct 18;14(10):e0223959. doi: 10.1371/journal.pone.0223959 (PMC6799915; doi:10.1371/journal.pone.0223959)
Supplement: S1 Table — During the collection day rats drank Tap Water with 5g/L of sucrose to increase the diuresis. Group A–administration of IP6Na12, Group B–administration of phytin. (PDF) [file pone.0223959.s001.pdf]

Table S1. Results of concentration and excretion determined by non-specific spectrometric quantification of InsPs. During the collection day rats drank Tap Water with 5g/L of sucrose to increase the diuresis. Group A – administration of $IP6Na_{12}$, Group B – administration of phytin.

GROUP A	*DAY 0*				*GROUP B*	*DAY 0*			
Rat	**[IPs] µM**	**SE**	**Exc IPs nmol/20h**	**SE**	***Rat***	**[IPs] µM**	**SE**	**Exc IPs nmol/20h**	**SE**
1	0.007		0.091		***1***	0.019		0.510	
2	0.060		0.622		***2***	0.077		0.963	
3	0.000	0.031	0.000	0.131	***3***	0.060	0.100	1.860	0.311
4	0.194		0.698		***4***	0.034		0.578	
5	0.000		0.000		***5***	0.639		1.917	
6	0.009		0.090		***6***	0.011		0.051	
Mean	0.047		0.320		***Mean***	0.138		0.910	
GROUP A	*DAY 7*				*GROUP B*	*DAY 7*			
Rat	**[IPs] µM**	**SE**	**Exc IPs nmol/20h**	**SE**	***Rat***	**[IPs] µM**	**SE**	**Exc IPs nmol/20h**	**SE**
1	-		-		***1***	0.027		1.107	
2	0.623		11.526		***2***	0.039		0.527	
3	0.451	0.047	7.893	1.279	***3***	0.091	0.055	3.549	0.537
4	0.667		5.203		***4***	0.323		2.778	
5	0.463		4.630		***5***	0.169		3.803	
6	0.448		5.376		***6***	0.320		2.624	
Mean	0.530		6.925		***Mean***	0.162		2.398	
GROUP A	*DAY 14*				*GROUP B*	*DAY 14*			
Rat	**[IPs] µM**	**SE**	**Exc IPs nmol/20h**	**SE**	***Rat***	**[IPs] µM**	**SE**	**Exc IPs nmol/20h**	**SE**
1	0.309		3.554		***1***	0.200		5.600	
2	0.318		4.611		***2***	0.378		5.859	
3	0.220	0.066	2.970	1.507	***3***	0.111	0.040	5.550	0.417
4	0.306		2.142		***4***	0.300		4.050	
5	0.205		2.870		***5***	0.168		4.704	
6	0.651		12.044		***6***	0.273		3.276	
Mean	0.335		4.698		***Mean***	0.238		4.840	
