# Supplementary material for: Intake of myo-inositol hexaphosphate and urinary excretion of inositol phosphates in Wistar rats: Gavage vs. oral administration with sugar
Source: PLoS One. 2019 Oct 18;14(10):e0223959. doi: 10.1371/journal.pone.0223959 (PMC6799915; doi:10.1371/journal.pone.0223959)
Supplement: S2 Table — During the collection day rats drank Tap Water with 5g/L of sucrose to increase the diuresis. Group A–administration of IP6Na12, Group B–administration of phytin. (PDF) [file pone.0223959.s002.pdf]

Table S2. Concentration and excretion values of InsP6 obtained by semiquantification with polyacrylamide gel electrophoresis (PAGE). During the collection day rats drank Tap Water with 5g/L of sucrose to increase the diuresis. Group A– administration of $IP6Na_{12}$, Group B– administration of phytin.

GROUP A	***DAY 14***				***GROUP B***	***DAY 14***			
Rat	**[IP_6] µM**	**SE**	**Exc IP_6 nm/20h**	**SE**	***Rat***	**[IP_6] µM**	**SE**	**Exc IP_6 nm/20h**	**SE**
1	0.043	0.038	0.492	0.727	***1***	0.016	0.002	0.445	0.053
2	0.030		0.435		***2***	0.018		0.275	
3	0.025		0.331		***3***	0.013		0.632	
4	0.119		0.831		***4***	0.024		0.327	
5	0.069		0.963		***5***	0.013		0.370	
6	0.266		4.929		***6***	0.027		0.322	
Mean	0.092		1.330		***Mean***	0.018		0.395	
