# Supplementary material for: Intake of myo-inositol hexaphosphate and urinary excretion of inositol phosphates in Wistar rats: Gavage vs. oral administration with sugar
Source: PLoS One. 2019 Oct 18;14(10):e0223959. doi: 10.1371/journal.pone.0223959 (PMC6799915; doi:10.1371/journal.pone.0223959)
Supplement: S3 Table — During the collection day rats drank Tap Water with 10 g/L of sucrose to increase the diuresis. GC–administration of IP6Na12, GD–administration of IP6Mg2Ca4, GE–IP6Na12 + Ca. (PDF) [file pone.0223959.s003.pdf]

Table S3. Results of concentration obtained by non-specific spectrometric quantification of InsPs. During the collection day rats drank Tap Water with 10 g/L of sucrose to increase the diuresis. GC – administration of $IP6Na_{12}$, GD – administration of $IP6Mg_2Ca_4$, GE – $IP6Na_{12}$ + Ca.

	DAY 0		***DAY 7***		***DAY 14***		***DAY 21***		***DAY 28***		***DAY 35***	
Rats GC	**[IPs] µM**	**SE**	**[IPs] µM**	**SE**	**[IPs] µM**	**SE**	**[IPs] µM**	**SE**	**[IPs] µM**	**SE**	**[IPs] µM**	**SE**
1	0.000		0.023		0.021		0.094		0.062		0.119	
2	0.031		0.035		0.022		0.144		0.068		0.124	
3	0.032	0.006	-	0.043	0.062	0.195	0.535	0.134	-	0.050	-	0.317
4	0.000		-		0.124		0.795		0.330		-	
5	0.004		0.004		0.248		0.842		0.183		1.069	
6	0.017		0.191		0.171		0.706		0.220		1.339	
Mean	0.014		0.063		0.216		0.519		0.173		0.663	
Rats GD	**[IPs] µM**	**SE**	**[IPs] µM**	**SE**	**[IPs] µM**	**SE**	**[IPs] µM**	**SE**	**[IPs] µM**	**SE**	**[IPs] µM**	**SE**
1	0.052		0.000		-		0.082		0.017		0.038	
2	0.075		0.000		0.000		0.084		0.022		0.047	
3	0.070	0.010	0.278	0.046	0.000	0.036	0.173	0.061	0.138	0.031	0.302	0.049
4	0.122		0.030		0.000		0.165		-		0.175	
5	0.078		0.000		0.000		0.153		0.112		0.129	
6	0.097		0.000		0.178		0.481		-		0.310	
Mean	0.082		0.051		0.036		0.190		0.072		0.167	
Rats GE	**[IPs] µM**	**SE**	**[IPs] µM**	**SE**	**[IPs] µM**	**SE**	**[IPs] µM**	**SE**	**[IPs] µM**	**SE**	**[IPs] µM**	**SE**
1	0.026		0.088		0.090		0.086		0.160		0.223	
2	0.119		0.059		0.678		0.000		0.668		1.301	
3	0.011	0.018	0.023	0.016	0.048	0.099	0.000	0.017	0.173	0.098	0.293	0.175
4	0.058		0.108		0.108		-		0.236		0.320	
5	0.000		-		0.074		0.000		0.160		0.351	
6	0.018		0.102		0.131		0.000		-		0.843	
Mean	0.039		0.076		0.188		0.017		0.279		0.555	
