# Supplementary material for: Intake of myo-inositol hexaphosphate and urinary excretion of inositol phosphates in Wistar rats: Gavage vs. oral administration with sugar
Source: PLoS One. 2019 Oct 18;14(10):e0223959. doi: 10.1371/journal.pone.0223959 (PMC6799915; doi:10.1371/journal.pone.0223959)
Supplement: S4 Table — During the collection day rats drank Tap Water with 10g/L of sucrose to increase the diuresis. GC–administration of IP6Na12, GD–administration of IP6Mg2Ca4, GE–IP6Na12 + Ca. (PDF) [file pone.0223959.s004.pdf]

Table S4. Excretion values obtained by non-specific spectrometric quantification of InsPs. During the collection day rats drank Tap Water with 10g/L of sucrose to increase the diuresis. GC – administration of $IP6Na_{12}$, GD – administration of $IP6Mg_2Ca_4$, GE – $IP6Na_{12}$ + Ca.

	DAY 0		***DAY 7***		***DAY 14***		***DAY 21***		***DAY 28***		***DAY 35***	
Rats GC	**Exc nmol /20h**	**SE**	**Exc nmol /20h**	**SE**	**Exc nmol /20h**	**SE**	**Exc nmol /20h**	**SE**	**Exc nmol /20h**	**SE**	**Exc nmol /20h**	**SE**
1	0.000		1.334		1.029		3.384		3.286		7.378	
2	0.775		1.190		1.034		3.456		3.060		6.324	
3	1.376	0.236	-	0.313	0.074	0.329	5.618	0.335	-	0.365	-	0.416
4	0.000		-		0.149		3.975		2,046		-	
5	0.220		0.027		0.298		3.873		1.665		5.345	
6	1.020		1.299		0.205		4.377		1.496		6.427	
Mean	0.565		0.963		0.465		4.114		2.311		6.369	
Rats GD	**Exc nmol /20h**	**SE**	**Exc nmol /20h**	**SE**	**Exc nmol /20h**	**SE**	**Exc nmol /20h**	**SE**	**Exc nmol /20h**	**SE**	**Exc nmol /20h**	**SE**
1	4.108		0.000		-		5.494		1.156		2.014	
2	2.550		0.000		0.000		4.032		1.474		1.551	
3	1.750	0.423	4.309	0.704	0.000	0.199	2.855	0.391	1.518	0.081	0.785	0.213
4	3.172		1.050		0.000		3.548		-		0.595	
5	2.574		0.000		0.000		3.596		1.344		1.355	
6	1.164		0.000		0.997		2.982		-		1.054	
Mean	2.553		0.893		0.199		3.751		1.373		1.226	
Rats GE	**Exc nmol /20h**	**SE**	**Exc nmol /20h**	**SE**	**Exc nmol /20h**	**SE**	**Exc nmol /20h**	**SE**	**Exc nmol 20h**	**SE**	**Exc nmol /20h**	**SE**
1	1.404		2.288		0.702		2.236		1.920		4.014	
2	2.559		0.502		3.526		0.000		3.741		5.724	
3	0.605	0.379	1.035	0.401	0.768	0.441	0.000	0.447	2.336	0.391	5.421	0.574
4	0.325		2.700		1.620		-		3.776		6.880	
5	0.000		-		1.850		0.000		2.320		3.721	
6	0.630		1.530		0.838		0.000		-		3.203	
Mean	0.920		1.611		1.551		0.447		2.818		4.827	
